# A National Pension Fund for Hospital Officials and Nurses

**Published:** 1887-05-21

**Authors:** 


### A National Pension Fund for Hospital Officials and Nurses.

### The Views of Officials, Sisters, Nurses, and others.

### By a Nurse of a Private Nursing Establishment.

With regard to the Pension Fund, I and all the nurses I have heard speak of it consider the proposal a most excellent one. We certainly consider that all nurses who have only received one year's training should be allowed to join. The rules of the hospital I was trained in (the Royal Infirmary) say we are trained at the end of one year, but they expect nurses to remain two years more, for the sake of having trained nurses for the hospital, but many like myself find hospital work too hard, and have to become private nurses. I would be glad to get all particulars. Nurses receive very small pay for the work they do, and often have many calls on that small pay. It would make a poor nurse's life very much happier if she had some little thing to look forward to, to help her at the age of fifty. I have heard many say they do not know what their end will be. Another great want (I think it has been spoken of) is a home hospital for nurses, when they are ill and run down. More than half of our nurses in homes and hospitals have no home—no father or mother; they go on for years working, each year getting more and more run down, and when they do break down altogether have nowhere to go. Hospitals and homes cannot keep them, and they simply have nowhere to rest their weary bodies except to throw themselves on the charity of relations who themselves have enough to do. The very worry of this only makes them worse. I have known cases of this kind. Is there any hope of such a home for sick nurses?

### By the late Assistant Matron of a London Infirmary.

Seeing you are open to receive the names of those who are anxious to see the establishment of a Pension Fund for nurses, I trouble you with this note. I have long been interested in the cause, and am sure that many nurses are. Some nurses who have worked for years in infirmaries are interested in the movement. I believe the co-operation might be gained of matrons and superintendents of nursing institutions, and I think many nurses would contribute, with the understanding that they would forfeit claim to the pension if nursing were given up either before they arrived at a certain age, or before they had worked a certain number of years, unless any accident arising from the profession incapacitated them.

